# The fish diversity in the upper reaches of the Salween River, Nujiang River, revealed by DNA barcoding

**DOI:** 10.1038/srep17437

**Published:** 2015-11-30

**Authors:** Weitao Chen, Xiuhui Ma, Yanjun Shen, Yuntao Mao, Shunping He

**Affiliations:** 1 The Key Laboratory of Aquatic Biodiversity and Conservation of Chinese Academy of Sciences, Institute of Hydrobiology, Chinese Academy of Sciences, Wuhan, Hubei, 430072, China; 2 Graduate school of Chinese Academy of Sciences, Beijing, 10001, China; 3 School of life science, Southwest University, Beibei, Chongqing, 400715, China

## Abstract

Nujiang River (NR), an essential component of the biodiversity hotspot of the Mountains of Southwest China, possesses a characteristic fish fauna and contains endemic species. Although previous studies on fish diversity in the NR have primarily consisted of listings of the fish species observed during field collections, in our study, we DNA-barcoded 1139 specimens belonging to 46 morphologically distinct fish species distributed throughout the NR basin by employing multiple analytical approaches. According to our analyses, DNA barcoding is an efficient method for the identification of fish by the presence of barcode gaps. However, three invasive species are characterized by deep conspecific divergences, generating multiple lineages and Operational Taxonomic Units (OTUs), implying the possibility of cryptic species. At the other end of the spectrum, ten species (from three genera) that are characterized by an overlap between their intra- and interspecific genetic distances form a single genetic cluster and share haplotypes. The neighbor-joining phenogram, Barcode Index Numbers (BINs) and Automatic Barcode Gap Discovery (ABGD) identified 43 putative species, while the General Mixed Yule-coalescence (GMYC) identified five more OTUs. Thus, our study established a reliable DNA barcode reference library for the fish in the NR and sheds new light on the local fish diversity.

The Nujiang River (NR), an important international river in southwestern China, is an essential component of the Mountains of Southwest China biodiversity hotspot (www.conservation.org). A rich biodiversity has developed in this region because of the complicated geological history and dramatic variations in local climates and topography^1^. Of the 77 known fish species distributed throughout the NR basin (the upper reaches of the Salween River), numerous species are endemic and even endangered (e.g., *Akrokolioplax bicornis* and *Garra cryptonemus*)^2^,^3^. In addition, exotic invasive species (e.g., *Abbottina rivularis, Oreochromis niloticus, Rhinogobius giurinus* and *Rhodeus ocellatus*) were imprudently introduced as economic fish^1^,^2^. Meanwhile, numerous new species have been discovered and described during the past several years^4^,^5^,^6^,^7^,^8^.

Freshwater ecosystems are being heavily exploited and are thus degraded by human activities around the world^9^,^10^, including in the NR, where fishes and fisheries are strongly affected. In recent decades, the fish in the NR have been severely threatened by activities including overfishing, mining, the construction of hydropower stations, and environmental disruption. Moreover, the impending construction of thirteen dam cascades along the Nujiang main stem in the coming years poses a new threat^11^ that will worsen the situation for the fish in the NR. Thus, currently, an understanding of the fish diversity in the NR appears to be particularly important.

With regard to the fish in the NR, the studies conducted to date have primarily focused on investigations of fish fauna based on morphological features, descriptions of new species, and population genetics or phylogenetic analyses of several species^2^,^5^,^8^,^12^,^13^,^14^,^15^. Therefore, the use of molecular techniques not only favorably complements traditional taxonomic methods but also provides a new perspective on fish diversity.

For the past several hundred years, taxonomic descriptions of species were largely accomplished through morphological characterizations^16^. However, misidentifications occurred because of features such as phenotypic plasticity, genotypic variation, cryptic species, or differing life stages. DNA barcoding, using a standardized molecular tag located in the mitochondrial cytochrome *c* oxidase I gene (COI), has become a dominant approach for the rapid and accurate discrimination of animal species^17^,^18^. The technology has drawn considerable attention and has demonstrated a high success rate in fish identifications, with mean levels of COI diversity within fish species of approximately 0.3–0.4%^19^. In addition, a few examples of deep intraspecific divergence in fish species indicate that cryptic speciation may be discovered when employing DNA barcoding^19^,^20^,^21^,^22^,^23^,^24^,^25^. DNA barcoding, therefore, not only enables accurate species delimitation but also flags the likely existence of morphologically cryptic species. DNA barcodes also have numerous other applications, e.g., the identification of fish parts or remnants (fish filets, eggs, and larvae)^26^,^27^,^28^, the tracking of exotic invasive species^29^, the enhancement of wildlife protection, and the protection of consumers from market fraud^27^. However, because mitochondrial DNA is maternally inherited, DNA barcoding has limitations in identifying species associated with incomplete lineage sorting, introgression hybridization, or ancestral polymorphism.

The main objectives of this study were (1) to test whether a species can be reliably identified with DNA barcoding, (2) to establish a DNA barcoding library for the ichthyofauna in the NR, (3) to provide new insights into fish diversity by employing several analytical methods, and (4) to reveal uncertainty in species in which discrepancies are observed between genetic data and morphological taxonomy.

## Results

**Figure 1:**
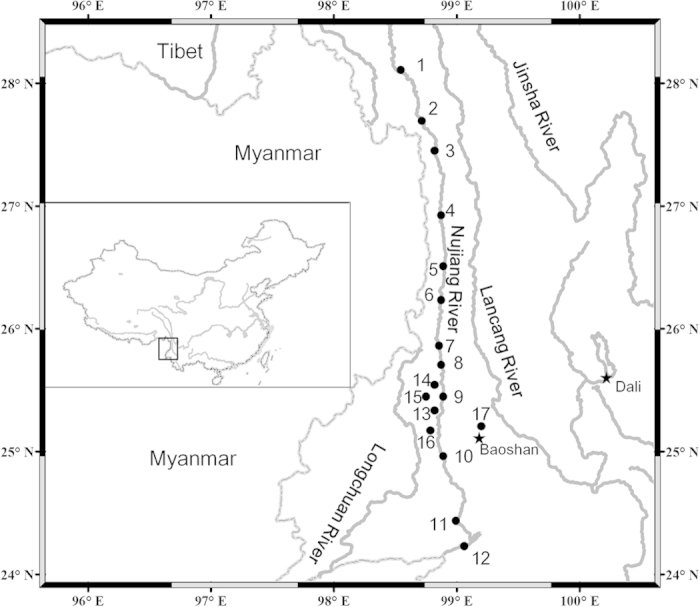
Map of locations sampled in this study. The numbers of the locations refer to 1) Bingzhongluo, 2) Puladi, 3) Maji, 4) County Areas of Fugong, 5) Pihe, 6) Chenggan, 7) Liuku, 8) Hongqi Dam, 9) Mangkuan, 10) Xiaopingtian, 11) Sanjiangkou, 12) Longzhen Bridge, 13) Kongguang Village, 14) Manglong River, 15) Mangkuan River, 16) Mangliu River, and 17) Longwang Pond. Locations 1–12 are sampling sites in the main stem, and 13–17 are located in a tributary. Location details and a list of the number of samples collected per site are provided in Supplementary Table S1. Map was created in the ArcGIS version 10.1 and modified in Microsoft Office.

We obtained mitochondrial barcodes (648 bp) for a total of 1139 fish specimens belonging to 5 orders, 10 families, 31 genera and 46 a priori identified species from 17 locations in the NR basin (Fig. 1). This list of 46 morphological species includes 16 endemic species, 9 exotic invasive species and 21 widespread species (Table S3). No deletions, insertions or stop codons were detected in any of the amplified sequences, demonstrating that all of the sequences constitute functional mitochondrial COI sequences. For the majority of species, multiple specimens (mean = 21.3 specimens per species) from distant localities were analyzed to document intraspecific divergence. Only three species were represented by a single specimen, and two species (*Schizothorax nukiangensis* and *Triplophysa nujiangen*) were represented by 226 and 107 individuals, respectively (Table S1).

**Figure 2:**
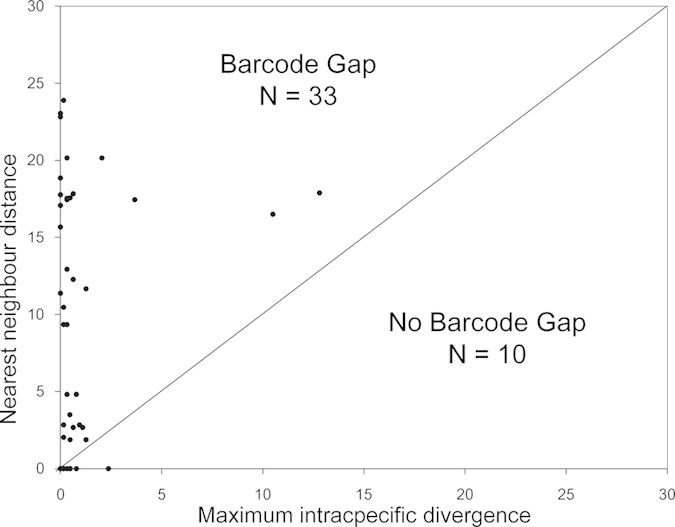
Maximum intraspecific divergence compared with the nearest-neighbor distance for fish in the Nujiang River. Only species with multiple sequences (Table S1) are presented. All of these species fall above the 1:1 line, indicating the presence of a ‘barcode gap.’

As expected, a hierarchical increase in the mean genetic variation from within species (mean = 0.41%, standard error [SE] = 0.000), to within congeners (mean = 2.14%, SE = 0.002), to within families (mean = 12.47%, SE = 0.006) was observed in the K2P model (Table 1). Overall, the genetic divergence among congeneric species was an average of approximately 5 times greater than that among individuals of the same species. The intraspecific divergence exhibited considerable heterogeneity and ranged from 0 to 12.80%, with a mean value of 0.41% (Table S3). Notably deep conspecific distances were observed for *Abbottina rivularis*, *Paramisgurnus dabryanus*, and *Rhodeus ocellatus*. Very low interspecific distances were observed among the two species in *Creteuchiloglanis*, three species in *Schizothorax*, and five species in *Schistura*. A regression analysis revealed that the intraspecific sequence divergence is not significantly correlated with the sample size (Spearman correlation analysis: P = 0.608). A barcoding gap analysis demonstrated that a barcode gap was present in 76.74% of all analyzed species (Fig. 2). For *Schizothorax gongshanensis*, *S. lissolabiatus* and *S. nukiangensis*; *Creteuchiloglanis gongshanensis* and *C. macropterus*; and *Schistura longa*, *S. poculi*, *S. prolixifasciata*, *S*. sp., and *S. vinciguerrae*, the distance to the nearest neighbor was zero.

**Figure 3:**
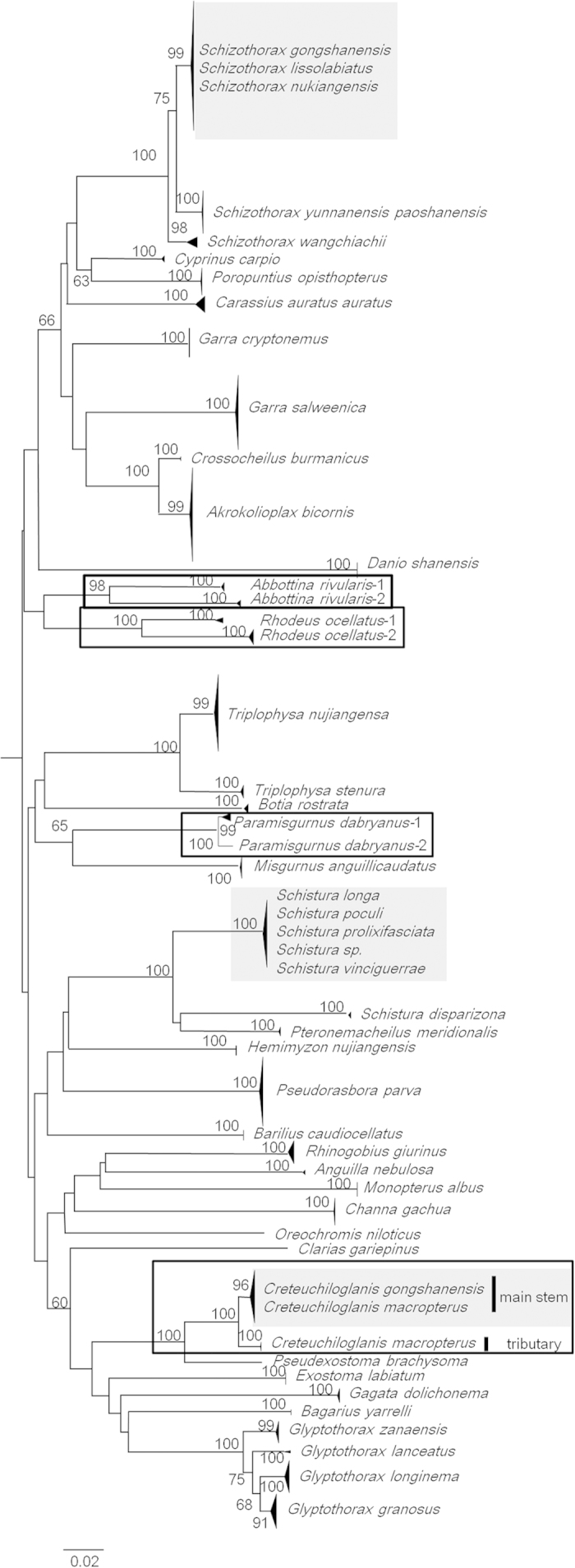
NJ tree of 1139 COI barcodes based on the K2P model. The framed clusters and the highlighted clusters in grey indicate species with a high cryptic diversity and species characterized by haplotype sharing or low interspecific distances, respectively.

The NJ tree derived from the complete barcode dataset contained 43 species clusters (including the singleton species) supported by bootstrap values of ≧91% (Fig. 3). Four species (*A. rivularis*, *C. macropterus*, *P. dabryanus*, and *R. ocellatus*) formed two distinct clusters supported by high bootstrap values (>98%), which indicates a high degree of cryptic diversity and possibly the occurrence of cryptic species (Fig. 3, S1, framed clusters). Within this group, *C. macropterus* was characterized by two distinct geographical splits, despite a low mean intraspecific divergence (0.52% K2P); one of these splits contained specimens from the main stem, and the other split contained specimens from the tributaries. Moreover, the NJ tree also revealed three cases of haplotype sharing between species (Fig. 3, S1, groups highlighted in grey). The lack of divergence observed in *Schistura* and *Schizothorax* is particularly intriguing because these genera involve three and five species, respectively, included in only one cluster per genus.

Of the 43 species, the character-based analyses successfully identified 33 species. Three newly designated species complexes (*Creteuchiloglanis* complex, *Schistura* complex, and *Schizothorax* complex) and three species with deep intraspecific divergence could also be recognized. Among the diagnosed species, six species were identified with a single nucleotide ND (nucleotide diagnostics; see methods), and the other species were diagnosed using an ND of two to three nucleotide positions in combination (Table 2).

**Figure 4:**
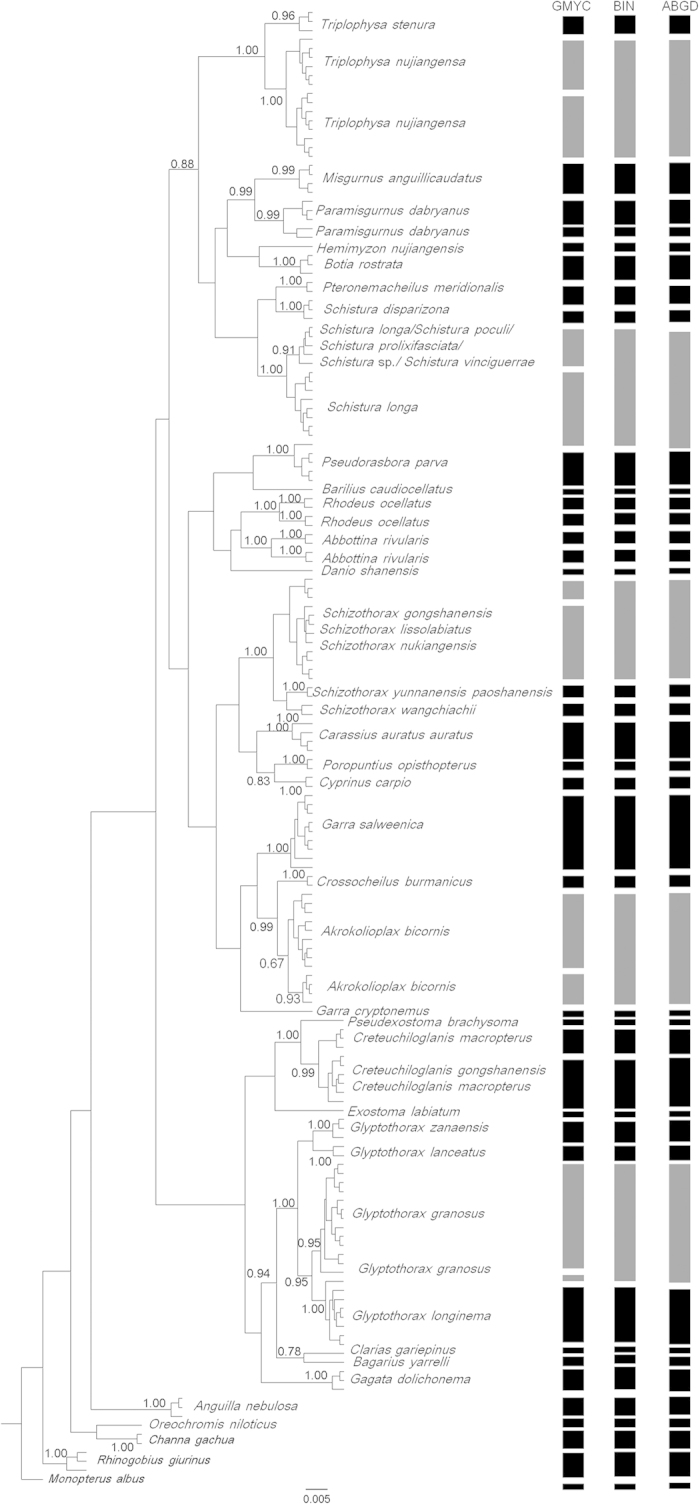
Bayesian inference gene tree with delineated OTUs. Grey rectangles represent species that share a COI lineage.

The BIN analysis led to the recognition of 43 OTUs (Fig. 4, Table S4). Twenty-six BIN clusters were found to be taxonomically concordant with the other barcode data that were BOLD-assigned to the same species name, while 16 BIN clusters were discordant with morphological species (Table S4). Moreover, one record (*Pseudexostoma brachysoma*) was indicated as a singleton, which means that this BIN only refers to one specimen that was not reported by BOLD. The count of OTUs produced by ABGD varied from 34 to 59 (Table S5). The ABGD analyses conducted with the JC69 and K2P models both produced two initial partitions with OTU counts of 34 (P = 0.0215–0.0599) and 43 (P = 0.0017–0.0129), respectively, whereas the use of the p distance returned 34 (P = 0.0215–0.0599) and 49 (P = 0.0017–0.0129) OTUs. The results obtained with the p distance were excluded because of the conflict with the results from other results with ABGD and those obtained with other analytical methods. Therefore, we chose the result of 43 OTUs because it was concordant with the outcome of both the BIN and NJ analyses (Fig. 4). Both the single- and multiple-threshold GMYC models outperformed the null model, indicating the presence of more than one species in the dataset (Table S6). The single-threshold model (48 OTUs) and the multiple-threshold model (50 OTUs) did not differ significantly from each other (χ2 = 5.11, d.f. = 8, 0.1 < P < 0.9). Thus, the outcome of the single-threshold model was adopted for further study. A comparison between the Bayesian inference (Fig. 4) and maximum-likelihood gene trees (Fig. S1) did not reveal obvious differences in the positioning of OTUs. The three methods yielded congruent results, but with five exceptions characterized by the assignment of OTUs to the PARTIAL MATCH category (highlighted in grey in Fig. 4). Both the BIN and ABGD analyses merged each of these five OTUs into a corresponding single OTU, whereas the GMYC approach partitioned each OTU into two OTUs.

**Figure 5:**
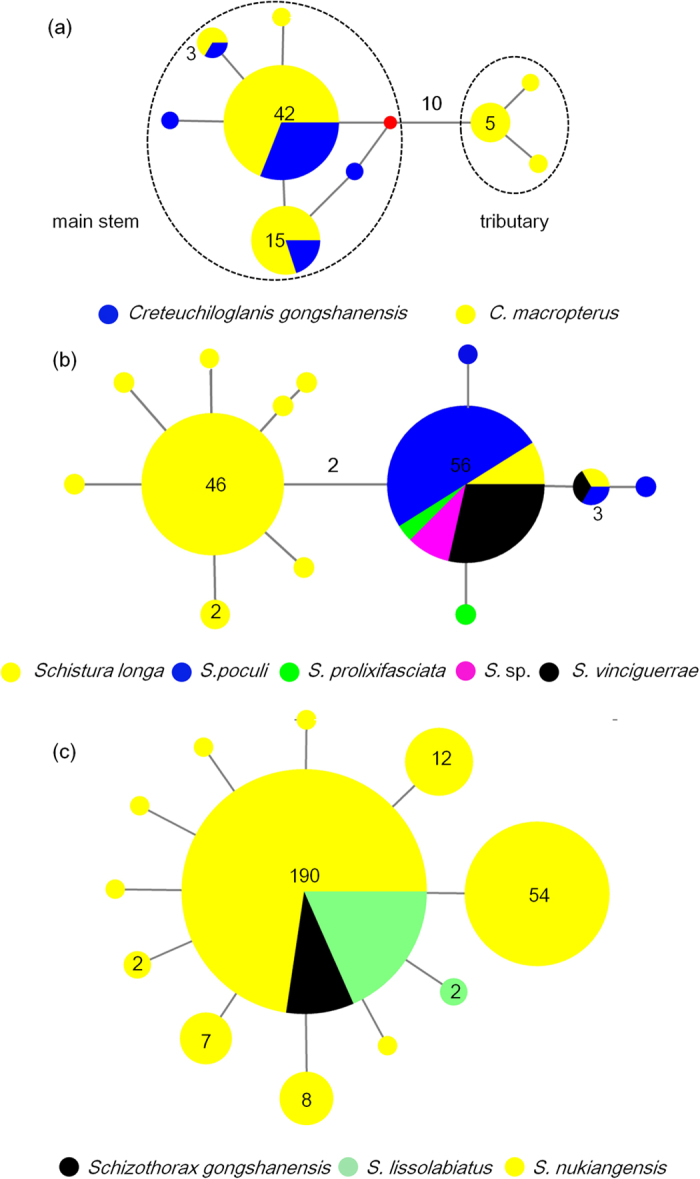
Haplotype networks of species with low interspecific divergences. (**a**) Genus Creteuchiloglanis; (**b**) genus Schistura; and (**c**) genus *Schizothorax*. The bold numbers on the internodes indicate mutation steps, and the other numbers are the frequencies of each haplotype. A red empty circle indicates missing intermediate steps between observed haplotypes.

The haplotype networks of the closely related species demonstrated that the sharing of COI haplotypes was common for three genera (*Creteuchiloglanis*, *Schistura*, and *Schizothorax*) (Fig. 5). The majority of the specimens of the three species in the genus *Schizothorax* shared a main haplotype. The genus *Creteuchiloglanis* generated two groups separated by 10 mutation steps; while one group contained specimens of *C. macropterus* from the tributary, the other included individuals of *C. gongshanensis* and *C. macropterus* from the main stem. Additionally, haplotype sharing was popular among *C. gongshanensis* and *C. macropterus* in the main stem. Five closely related species in *Schistura* formed two groups as well; one group only contained *Schistura longa*, and the other group consisted of five species (*Schistura longa*, *S. poculi*, *S. prolixifasciata*, *S*. sp., and *S. vinciguerrae*).

**Figure 6:**
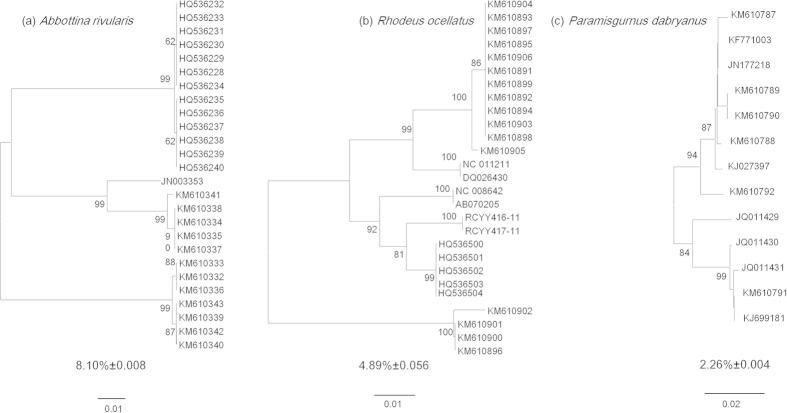
NJ trees and mean conspecific distances of the three species with deep intraspecific divergences based on the K2P model for our COI sequences and the downloaded sequences. (**a**) *Abbottina rivularis*; (**b**) *Rhodeus ocellatus*; and (**c**) *Paramisgurnus dabryanus*.

The ML and BI trees that were built based on the cytb dataset positioned the two reference species clustered closely with the species identification published on the NCBI website, which confirms the accuracy of our morphological identification; in addition, six and five clades were generated within *A. rivularis* (Fig. S2a) and *R. ocellatus* (Fig. S2b), respectively. Similarly, multiple lineages with deep conspecific divergences were detected in the NJ tree and evident from the higher genetic distances of the three species (*A. rivularis*, *R. ocellatus*, and *P. dabryanus*) based on the analysis of the combined COI dataset (Fig. 6).

## Discussion

Our study represents the first comprehensive molecular assessment of the fish in the NR, including the collection and analysis of approximately 60% of the currently known species (excluding Asian carp species). In the current study, DNA barcoding was effective in identifying species and provided a straightforward identification system when a perfect match existed between the morphology-based taxonomy and genetic divergence. Furthermore, the levels of haplotype sharing detected for three genera and the high intraspecific divergences observed for three invasive species demonstrate the need for further taxonomic research on these species. Overall, this study demonstrated the ability of DNA barcoding to help calibrate the current taxonomic resolution and to shed new light on the fish diversity in the NR basin.

The corrected mean level of intraspecific divergence of 0.41% (SE = 0.000) calculated for the NR fish is slightly greater than the values reported in several previous fish barcoding studies^16^,^30^,^31^,^32^, but is lower than those reported for North America’s freshwater fish, rainbow fish, and coral-reef fish, which may be explained by the effects of environmental homogeneity and frequent gene flow^24^,^25^,^26^.

At first glance, the analysis of COI sequences using the K2P model with the genetic distance and topology created by the NJ tree discriminated all 33 previously identified species. However, three species (*A. rivularis*, *R. ocellatus*, and *P. dabryanus*) displayed deep divergence (>1.7%), and two clades were not identified by the K2P model. With regard to these other unidentified species, *C. macropterus* individuals derived from the main stem shared a lineage and haplotype with *C. gongshanensis*. This pattern could be interpreted as recent speciation, as interspecific hybridization, or as misidentification^33^,^34^. To evaluate this phenomenon, population analyses should be performed with larger sample sizes and should employ additional molecular approaches to study the relationship between these two populations. In contrast, the character-based approach yielded a higher identification accuracy and successfully diagnosed 33 of the 43 morphological species (excluding the three singleton species). In addition, the three species that failed to be identified by the former two techniques could be delimited by the character-based approach. Furthermore, the BIN and ABGD analytical methods successfully delineated 33 species, while the GMYC method performed slightly worse and diagnosed only 30 species. All of the analytical methods employed in our study failed to recognize the two species in *Creteuchiloglanis*, three species of *Schizothorax*, and the five species of *Schistura* because of the absence of interspecific diversity.

For our dataset, the barcode gap analysis failed for 10 species (in three genera) in which the distance to the nearest neighbor is zero. Haplotype sharing is common in schizothoracine fishes^12^,^13^. Thus, to date, phylogenetic and population studies that have focused on schizothoracine fish have demonstrated that many different species or subspecies in the same river basin shared mtDNA haplotypes, e.g., *Gymnocypris eckloni eckloni* and *G. e. scoliostomus* in the Yellow River system^35^ and *Schizothorax lissolabiatus* and *S. nudiventris* in the Mekong River basin^13^.

The ABGD and BIN methods produced identical results and delineated 43 OTUs, whereas the GMYC approach defined a greater number of OTUs (48 OTUs). The additional OTUs identified by the GMYC method were *Akrokolioplax bicornis*, *Glyptothorax granosus*, *Triplophysa nujiangensa*, a *Schistura* complex, and a *Schizothorax* complex. Each species or species complex formed two OTUs, which potentially implies detectable intraspecific diversity. Accordingly, the further study of conspecificity by, for example, population genetics research should be performed in the future to evaluate intraspecific divergence in detail.

The ABGD method generates diverse outcomes, and it is difficult to select the most appropriate outcome. The adoption of a single value of P = 0.01 has been proposed because this value was demonstrated to produce the strongest congruence with previous studies when examining the same data using multiple approaches^36^. In our study, the number of OTUs with P = 0.01 based on both the K2P and JC69 distances was strongly concordant with those obtained using other approaches. In contrast, while the GMYC approach has a strong theoretical basis, it typically generates a greater number of OTUs than other methods^37^,^38^,^39^,^40^, as evidenced by the definition of five additional OTUs in our study using the GMYC method. Compared with the two above-mentioned methods, the BIN method is not only the most rapid method, but it is also the only method that delivers one certain result. In our study, the BIN method produced an outcome in accordance with those based on the ABGD method and the NJ trees based on distance metrics.

In our study, the cases of low genetic divergence or haplotype sharing involved 10 native species from 3 genera. The accuracy of DNA barcoding depends, in particular, on the extent of separation between intraspecific variation and interspecific divergence^41^. Therefore, the inability to differentiate these species is attributed to a lack of genetic divergence among these species. In general, interspecific haplotype sharing has four possible explanations: hybridization, incomplete lineage sorting, inadequate taxonomy, and erroneous identification^33^. In the genus *Schizothorax*, interspecific haplotype sharing is a ubiquitous pattern in the same drainage^12^,^13^,^42^. First, species living in the same river drainage with a large distribution range may exhibit morphological variations^43^, and the shape of the mouth and lips (important characteristics used for fishes’ diets and species identification^44^) can vary depending on the developmental stages of individuals^45^, which may result in a lack of consensus regarding their taxonomy and lead to misidentification. Second, the pattern may also be attributed to rapid radiation^12^. Because only sparse investigations have been conducted, little is known regarding the other two genera and further studies are needed to interpret the pattern. Thus, in order to disentangle the relationships among these closely related species, more detailed studies (e.g., more detailed morphological analyses and population-level analyses with larger sample size) should be employed to these species in the future.

At the other end of the spectrum, the presence of multiple clusters or OTUs with deep divergences observed in three invasive species was indicative of cryptic diversity. Furthermore, previous phylogenetic studies on *A. rivularis*^46^ and *R. ocellatus*^47^ also reported an unexpectedly high degree of genetic diversity. In fact, cryptic diversity is typical in several non-economic fish species^48^,^49^. Deep genetic divergences within nominal species can be interpreted as misidentification and, more importantly, as cryptic or unrecognized speciation events^23^,^24^,^25^,^50^. Recognizing cryptic diversity has indisputably increased our knowledge regarding the biodiversity of numerous taxa, including fish^26^,^51^. Unexpectedly, the high divergence displayed within *A. rivularis*, *R. ocellatus* and *P. dabryanus* in this river drainage could be explained by their introductions from different sources. Furthermore, multiple lineages generated through trees based on the combined sequence data in our study demonstrated the potential occurrence of sources from other drainages within the three species. In the *P. dabryanus*, additionally, hybridization between *P. dabryanus* and *Misgurnus anguillicaudatus* in the natural range may also increase the genetic diversity and even contribute to the difficulty in obtaining an accurate identification^52^. With regard to the *C. macropterus*, two observed geographical lineages are very likely to result from the long-term geographical isolation.

## Methods

### Ethics statement

The methods involving animals in this study were conducted in accordance with the Laboratory Animal Management Principles of China. All experimental protocols were approved by the Ethics Committee of the Institute of Hydrobiology, Chinese Academy of Sciences.

### Samples and laboratory analyses

For this study, sample collections were performed in the NR basin in Yunnan province because the majority of the reported species are present there. A total of 1139 specimens were collected during March and October 2012 and May and June 2013 from localities in the upper-middle NR (Fig. 1). The sampling map was generated using the ArcGIS and modified in Microsoft Office. A small piece of white muscle tissue or fin was dissected from the right side of the body of each specimen. All of the tissue samples used for genomic DNA extraction were preserved in 95% ethanol and deposited in the Freshwater Fish Museum at the Institute of Hydrobiology, Chinese Academy of Sciences. Vouchers were morphologically identified to identification reliability level two, as described by the Fish-BOL collaborators’ protocol^53^, namely, ‘specimen identified by a trained identifier who had prior knowledge of the group in the region or used available literature to identify the specimen’. References in the literature used in our study are as follows: (Chu and Chen, 1989; Zhu, 1989; Chen, 1998; Maurice, 2003; Zhou and Kottelat, 2005; Ng *et al.*, 2012; Jiang *et al.*, 2012)^1^,^2^,^3^,^4^,^5^,^54^,^55^,^56^.

Total genomic DNA was extracted from the muscle tissue or fin by performing a standard salt extraction. The COI barcode region was amplified using the universal primers FishF1 and FishR1^31^. The polymerase chain reaction (PCR) contained approximately 100 ng of template DNA, 1 μl of each primer (10 pmol), 3 μl of 10× reaction buffer, 1.5 μl of dNTPs (2.5 mM each), and 2.0 U of Taq DNA polymerase in a total volume of 30 μl. The PCR conditions for COI included an initial denaturation step at 95 °C for 5 min; followed by 30 cycles of denaturation at 95 °C for 1 min, annealing at 50 °C for 45 seconds, and extension at 72 °C for 45 seconds; followed by a final extension at 72 °C for 10 min. The amplified DNA was fractionated by electrophoresis in 1% low-melting-temperature agarose gels. However, because of the presence of mixed peaks in the PCR product of *Pseudexostoma brachysoma* using the selected primers, we cloned the PCR product using the pMD18-T Easy Vector System. PCR products and clones of expected sizes were sequenced directly. The aligned sequences were submitted to GenBank (accession numbers: KM610332–KM611465 and KC871095–KC871099), and the sequences, trace files, and specimen data were submitted to BOLD (Table S1).

### Data analyses

Bidirectional sequencing was employed to decrease the occurrence of sequencing mistakes. The COI sequences were initially edited using the DNASTAR multiple package (DNASTAR Inc., Madison, WI, USA) and aligned using the CLUSTALX 2.0 program^57^. The noisy sequences of both ends were trimmed before subsequent sequence analyses. We used distance-, tree-, and character-based DNA barcoding methods for species discrimination. Genetic distances within species, genera, and families, determined using the Kimura 2-parameter (K2P)^58^ and p distance, were inputted into the MEGA 4.1 program^59^. To examine the barcode gap, species-level comparisons between the maximum intraspecific genetic distance and the minimum distance to the nearest neighbor were performed using the ‘Barcoding Gap Analysis’ tool in BOLD^60^, and three singleton species were excluded. A regression analysis was performed to assess the relationship between species size and the mean intraspecific divergence. Neighbor-joining (NJ) trees based on the K2P and the p distance were implemented in MEGA 4.1 using 1000 bootstrap replicates to assess the branch support. However, we found that the differences in genetic distance and tree topology between the two trees (K2P and p distance) were minimal. Consequently, only the K2P model was chosen for tree reconstruction because it is commonly employed to evaluate phylogenetic relationships between species in DNA barcoding studies^18^,^61^. Finally, we used BLOG 2.0 to perform a character-based identification method with default parameters for species represented by greater than two individuals^62^,^63^,^64^. This method is mainly based on only the nucleotides of the specific sites in particular taxa for species diagnosis, and these diagnostic sites are referred to as nucleotide diagnostics (ND)^65^,^66^.

To group specimens into OTUs and further delimit species, three popular approaches, namely, Barcode Index Numbers (BINs)^67^, *Automatic Barcode Gap Discovery* (ABGD)^36^ and the General Mixed Yule-coalescent (GMYC)^68^,^69^ approach, were applied. The first two methods form OTUs based on genetic distances using different clustering algorithms, whereas the GMYC approach is a model-based likelihood method that seeks to determine the threshold between speciation and coalescent events from an ultrametric gene tree. Sequences were automatically assigned to a BIN using the BOLD Workbench v3.6 application (http://www.boldsystems.org; analyses performed on 23 December 2014). The ABGD analyses were performed on a web interface (wwwabi.snv.jussieu.fr/public/abgd/) using the default value for the relative gap width (X = 1.5) and both available distance metrics [JC69 and K2P], as well as the p-distance. While the default values were employed for the other parameter values, all of the assignments for intraspecific divergence (P) values between 0.001 and 0.100 were recorded. The GMYC method requires a fully resolved, ultrametric gene tree as input for the analysis. Therefore, we constructed a Bayesian inference tree in BEAST^70^ by employing a Yule pure-birth model^71^ tree with the following settings: GTR + I + G substitution model, empirical base frequencies, four gamma categories, and all codon positions partitioned with unlinked base frequencies and substitution rates. An uncorrelated relaxed lognormal clock model was employed with the rate estimated from the data and ucld-mean parameter with a uniform prior to value of 0 as a lower boundary and 10 as an upper boundary. All of the other settings were the default values. The length of the MCMC chain was 40 million, with sampling every 1000. The analyses were repeated twice, and convergence was assessed using the Tracer v1.5 application. The maximum clade credibility trees, with a 0.5 posterior probability limit, and the node heights of the target tree were constructed using TreeAnnotator v1.7.1. Both the single- and multiple-threshold GMYC analyses were conducted on the GMYC web server (http://species.h-its.org/gmyc/). The GMYC analyses were performed with the haplotype data, which was collapsed using ALTER^72^. A maximum-likelihood (ML) analysis was also performed with haplotype data to compare the results of Bayesian inference using RAXML-VI-HPC^73^ with the GTR + I + G model and 1000 nonparametric bootstrap replicates. The results of the BIN, ABGD and GMYC analyses were compared, and all of the OTUs were divided into three categories (FULL MATCH, i.e., all methods provide identical results; PARTIAL MATCH, i.e., two of the three methods delineated the same OTU; and DISCORDANT, i.e., the three methods all produced conflicting results) following the procedure introduced by Kekkonen and Hebert, 2014^74^.

In our study, species with low interspecific divergence were examined by using statistical parsimony networks^75^ to construct the relationship between haplotypes. These networks were constructed using the default 95% connection limit in the TCS 1.21 application^76^. To further infer the cryptic diversity of the species with deep intraspecific divergences, two approaches were employed. First, we amplified the mitochondrial cytochrome *b* gene (cytb) from the fish parts of individuals collected for our study (accession numbers: KP645235-KP645255) and downloaded the cytb sequences of species in the two genera (*Abbottina* and *Rhodeus*) from the NCBI database to infer the level of cryptic diversity and confirm the accuracy of the identification of the two reference species (*Abbottina rivularis* and *Rhodeus ocellatus*). The NJ trees based on the K2P distance were implemented in MEGA 4.1 using 1000 bootstrap replicates. Secondly, we combined our COI sequences with sequences downloaded from the BOLD (http://www.boldsystems.org/) or NCBI databases to infer cryptic diversity by generating a NJ tree and calculating the K2P distances using the MEGA 4.1 application. The GenBank accession numbers of the downloaded sequences are listed in Table S2.

## Additional Information

**How to cite this article**: Chen, W. *et al.* The fish diversity in the upper reaches of the Salween River, Nujiang River, revealed by DNA barcoding. *Sci. Rep.* **5**, 17437; doi: 10.1038/srep17437 (2015).

## Supplementary Material

Supplementary Information

Supplementary Dataset 1

## Figures and Tables

**Table 1:** Mean values, ranges, and standard deviations of genetic divergences, sorted by taxonomic level.

	Comparisons	Min%	Mean%	Max%	SE
Within Species	45141	0	0.41	12.80	0.000
Within Genus	35932	0	2.14	14.72	0.002
Within family	169727	2.85	12.47	30.29	0.006

**Table 2:** NDs for each species are listed along with sample sizes.

Species (sample sizes)	NDs	Species (sample sizes)	NDs
*Abbottina rivularis* (12)	A-300 + G-387	*Glyptothorax zanaensis* (17)	A-399 + G-468
*Akrokolioplax bicornis* (73)	G-252 + A-501	*Hemimyzon nujiangensis* (2)	A-387 + G-525
*Anguilla nebulosa* (4)	G-456 + C-525	*Misgurnus anguillicaudatus* (17)	G-468 + C-540
*Bagarius yarrelli* (4)	G-387 + A-399	*Monopterus albus* (11)	G-540
*Barilius caudiocellatus* (8)	G-486 + C-501	*Oreochromis niloticus* (1)	–
*Botia rostrata* (7)	T-468 + A-501	*Paramisgurnus dabryanus* (6)	A-468 + T-516 + T-525
*Carassius auratus auratus* (13)	T-456 + A-501	*Poropuntius opisthopterus* (21)	T-417
*Channa gachua* (21)	T-501 + T-540	*Pseudoexostoma brachysoma* (1)	–
*Clarias gariepinus* (1)	–	*Pseudorasbora parva* (54)	G-153
*Creteuchiloglanis* complex (78)	T-327 + A-402	*Pteronemacheilus meridionalis* (7)	G-312 + A-417 + T-456
*Crossocheilus burmanicus* (3)	G-402 + A-456	*Rhinogobius giurinus* (15)	G-288 + T-525
*Cyprinus carpio*(5)	G-399 + G-501	*Rhodeus ocellatus* (16)	A-501 + A-525
*Danio shanensis* (12)	T-300 + G-516	*Schistura* complex (116)	A-252 + G-387 + C-501
*Exostoma labiatum* (9)	A-264	*Schistura disparizona* (5)	G-318 + C-402
*Gagata dolichonema* (11)	T-168	*Schizothorax* complex (280)	C-399 + C-540
*Garra cryptonemus* (47)	A-321 + C-339 + A-402	*Schizothorax wangchiachii* (8)	C-387 + T-501
*Garra salweenica* (58)	C-486	*Schizothorax yunnanensis paoshanensis* (33)	A-327 + T-402 + G-516
*Glyptothorax granosus* (27)	C-252 + T-484	*Triplophysa nujiangensa* (107)	C-168 + C-484 + C-525
*Glyptothorax lanceatus* (2)	A-300 + G-321	*Triplophysa stenura* (10)	T-334 + C-375
*Glyptothorax longinema* (25)	T-318 + G-468		

The numbers provided in brackets are the sample sizes for each species.
