# Supplementary material for: The fish diversity in the upper reaches of the Salween River, Nujiang River, revealed by DNA barcoding
Source: Sci Rep. 2015 Nov 30;5:17437. doi: 10.1038/srep17437 (PMC4663501; doi:10.1038/srep17437)

**Supplementary information:**

**The fish diversity in the upper reaches of the Salween River, Nujiang River, revealed by DNA barcoding**

Weitao Chen^1,2^, Xiuhui Ma^3^, Yanjun Shen^1,2^, Yuntao Mao^1,2^ & Shunping He^1^*

^1^The Key Laboratory of Aquatic Biodiversity and Conservation of Chinese Academy of Sciences, Institute of Hydrobiology, Chinese Academy of Sciences, Wuhan, Hubei, 430072, China, ^2^Graduate school of Chinese Academy of Sciences, Beijing, 10001, China, ^3^School of life science, Southwest University, Beibei, Chongqing, 400715, China.

*Corresponding author

Corresponding author Phone: +862768780430; E-mail addresses: [clad@ihb.ac.cn](mailto:clad@ihb.ac.cn).

**Table S1:** Sample ID, sample location, BOLD Process ID, and GenBank accession numbers for each specimen.

**Table S2:** GenBank accession numbers of the downloaded sequences included in our study.

**Table S3:** Intraspecific genetic distance and nucleotide diversity of each species. (a) Intraspecific genetic distance (%) of each species. N: sample size of each species; the numbers 1, 2, and 3 in the bracket represent exotic species, native species, and endemic species, respectively.

**Table S4:** Detailed results of BINs from the “BIN Discordance Report” applied to the “NJBA” dataset using BOLD. The BINs are sorted according to the rank of conflict ranging from phylum (top) to species (bottom) level. The grey, light blue, and yellow highlights represent Discordance BINs, Concordance BINs, and Singletons, respectively.

**Table S5:** Results of the Automatic Barcode Gap Discovery (ABGD) analyses. X, relative gap width; Simple, p-distance; JC69, Jukes-Cantor substitution model; K2P, Kimura 2-parameter substitution model.

**Table S6:** Results of the General Mixed Yule-coalescent (GMYC) analyses. Clusters, OTUs delineated by the GMYC model with multiple specimens; Entities, singleton OTUs delineated by GMYC; CI, confidence interval; Likelihood null, likelihood of the null model; Likelihood GMYC, likelihood of the GMYC model; Threshold, the threshold between the speciation and coalescence processes; Single, single-threshold model; Multiple, multiple-threshold model; *** P < 0.001.

**Figure S1:** Maximum-likelihood tree with bootstrap values. The framed clusters and the clusters highlighted in grey indicate species with a high cryptic diversity and species characterized by haplotype sharing or low interspecific distances, respectively.

**Figure S2:** Tree resulting from the maximum likelihood analysis of Abbottina rivularis and Rhodeus ocellatus based on the cytb gene dataset. Bootstrap values are based on NJ analyses, and GenBank numbers represent novel sequences in our study.

Table S2

|  | COI (Genbank nos) |
| --- | --- |
| *Paramisgurnus dabryanus* | KF771003, KJ027397, KJ699181, JN177218, JQ011429-JQ011431 |
| *Abbottina rivularis* | JN003353, HQ536228-HQ536240 |
| *Rhodeus ocellatus* | NC_008642, NC_011211, DQ026430, AB070205, HQ536500-HQ536504, RC0572-RC0573(Process ID on BOLD) |
|  | cytb (Genbank nos) |
| *A. rivularis* | AY953020, JX137487-JX137687, EU934493-EU934497, EU934499, EU241412, KM261773 |
| *R. ocellatus/R. o. ocellatus* | AB769513-AB769519, HQ113265-HQ113266, AB109000, AB109009-AB109010, AF051876, DQ026430 |
| *R. o. kurumeus* | AB070205, AB366504-AB366507, AB769511-AB769512 |

Table S3

| Species | N | Min | Mean | Max |
| --- | --- | --- | --- | --- |
| *Abbottina rivularis*(1) | 12 | 0 | 6.75 | 12.80 |
| *Akrokolioplax bicornis*(3) | 73 | 0 | 0.15 | 0.93 |
| *Anguilla nebulosa*(2) | 4 | 0 | 0.16 | 0.31 |
| *Bagarius yarrelli*(2) | 4 | 0 | 0 | 0 |
| *Barilius caudiocellatus*(2) | 8 | 0 | 0 | 0 |
| *Botia rostrata*(2) | 7 | 0 | 0.13 | 0.47 |
| *Carassius auratus auratus*(1) | 13 | 0 | 0.47 | 1.25 |
| *Channa gachua*(2) | 21 | 0 | 0.02 | 0.15 |
| *Clarias gariepinus*(1) | 1 | - | - | - |
| *Creteuchiloglanis gongshanensis*(3) | 19 | 0 | 0.1 | 0.47 |
| *Creteuchiloglanis macropterus*(2) | 59 | 0 | 0.52 | 2.37 |
| *Crossocheilus burmanicus*(2) | 3 | 0 | 0.1 | 0.16 |
| *Cyprinus carpio*(2) | 5 | 0 | 0.12 | 0.31 |
| *Danio shanensis*(2) | 12 | 0 | 0 | 0 |
| *Exostoma labiatum*(2) | 9 | 0 | 0 | 0 |
| *Gagata dolichonema*(3) | 11 | 0 | 0.11 | 0.31 |
| *Garra cryptonemus*(3) | 47 | 0 | 0 | 0 |
| *Garra salweenica*(3) | 58 | 0 | 0.07 | 0.62 |
| *Glyptothorax granosus*(3) | 27 | 0 | 0.31 | 1.25 |
| *Glyptothorax lanceatus*(3) | 2 | 0.62 | 0.62 | 0.62 |
| *Glyptothorax longinema*(2) | 25 | 0 | 0.15 | 0.47 |
| *Glyptothorax zanaensis*(2) | 17 | 0 | 0.14 | 0.47 |
| *Hemimyzon nujiangensis*(3) | 2 | 0 | 0 | 0 |
| *Misgurnus anguillicaudatus*(2) | 17 | 0 | 0.06 | 0.31 |
| *Monopterus albus*(2) | 11 | 0 | 0 | 0 |
| *Oreochromis niloticus*(1) | 1 | - | - | - |
| *Paramisgurnus dabryanus*(1) | 6 | 0 | 1.75 | 3.67 |
| *Poropuntius opisthopterus*(3) | 21 | 0 | 0.08 | 0.16 |
| *Pseudexostoma brachysoma*(3) | 1 | - | - | - |
| *Pseudorasbora parva*(1) | 54 | 0 | 0.2 | 0.62 |
| *Pteronemacheilus meridionalis*(2) | 7 | 0 | 0.04 | 0.16 |
| *Rhinogobius giurinus*(1) | 15 | 0 | 0.46 | 2.04 |
| *Rhodeus ocellatus*(1) | 16 | 0 | 3.97 | 10.49 |
| *Schistura disparizona*(3) | 5 | 0 | 0.19 | 0.31 |
| *Schistura longa*(3) | 60 | 0 | 0.11 | 0.78 |
| *Schistura poculi*(2) | 30 | 0 | 0.03 | 0.47 |
| *Schistura prolixifasciata*(3) | 4 | 0 | 0.16 | 0.31 |
| *Schistura sp.*(2) | 5 | 0 | 0 | 0 |
| *Schistura vinciguerrae*(2) | 17 | 0 | 0.02 | 0.16 |
| *Schizothorax gongshanensis*(2) | 17 | 0 | 0 | 0 |
| *Schizothorax lissolabiatus*(2)  *Schizothorax nukiangensis*(3) | 37 | 0 | 0.02 | 0.15 |
|  | 226 | 0 | 0.11 | 0.47 |
| *Schizothorax wangchiachii*(1) | 8 | 0 | 0.27 | 1.09 |
| *Schizothorax yunnanensis paoshanensis*(3) | 33 | 0 | 0.07 | 0.16 |
| *Triplophysa nujiangensa*(3) | 107 | 0 | 0.21 | 0.78 |
| *Triplophysa stenura*(2) | 10 | 0 | 0.14 | 0.31 |

Table S4

| **Identification** | **Conflicting Taxon in BIN** | **Rank of Conflict** | **BIN** | **BIN Total Members** | **BIN Tax Variation** |
| --- | --- | --- | --- | --- | --- |
| ***Oreochromis niloticus*** | ***Cichlidae*** | Family | BOLD:ACR5811 | 188 | Cichlidae[168], Lutjanidae[2] |
| ***Carassius auratus auratus*** | ***Carassius*** | Genus | BOLD:AAA7176 | 320 | Carassius[317], Cyprinus[2] |
| ***Glyptothorax granosus*** | ***Glyptothorax*** | Genus | BOLD:ACC0606 | 30 | Glyptothorax[28], Gagata[2] |
| ***Paramisgurnus dabryanus*** | ***Paramisgurnus*** | Genus | BOLD:AAD6923 | 12 | Paramisgurnus[6], Misgurnus[4] |
| ***Rhinogobius giurinus*** | ***Rhinogobius*** | Genus | BOLD:AAI0449 | 37 | Rhinogobius[31], Acentrogobius[6] |
| ***Schistura longa Schistura poculi Schistura prolixifasciata***  ***Schistura vinciguerrae***  ***Schistura sp.*** | ***Schistura*** | Genus | BOLD:ACM0131 | 279 | Schistura[258], Pteronemacheilus[21] |
| ***Triplophysa nujiangensa*** | ***Triplophysa*** | Genus | BOLD:ACN9228 | 111 | Triplophysa[107], Claea[4] |
| ***Anguilla nebulosa*** | ***Anguilla nebulosa*** | species | BOLD:AAJ2664 | 14 | *Anguilla bengalensis bengalensis*[6], *Anguilla nebulosa*[4], *Anguilla nebulosa nebulosa*[1], *Anguilla bengalensis labiata*[1] |
| ***Clarias gariepinus*** | ***Clarias gariepinus*** | species | BOLD:ACF4787 | 47 | *Clarias gariepinus[*45], *Clarias sp*. NM2010[2] |
| ***Creteuchiloglanis gongshanensis***  ***Creteuchiloglanis macropterus*** | ***Creteuchiloglanis gongshanensis Creteuchiloglanis macropterus*** | Species | BOLD:AAX0831 | 64 | *Creteuchiloglanis macropterus*[44], *Creteuchiloglanis gongshanensis*[19], *Creteuchiloglanis gongshanensi*[1] |
| ***Cyprinus carpio*** | ***Cyprinus carpio*** | Species | BOLD:AAA7175 | 219 | *Cyprinus carpio*[178], *Cyprinus carpio carpio*[17], *Cyprinus carpio x Procypris rabaudi*[2], *Cyprinus pellegrini*[1], *Cyprinus multitaeniata*[1] |
| ***Misgurnus anguillicaudatus*** | ***Misgurnus anguillicaudatus*** | Species | BOLD:ACB4634 | 34 | *Misgurnus anguillicaudatus*[30], *Misgurnus sp. AL8*[1], *Misgurnus sp. AL7*[1], *Misgurnus sp. AF12*[1], *Misgurnus sp.* *AF10*[1] |
| ***Poropuntius opisthopterus*** | ***Poropuntius opisthopterus*** | Species | BOLD:AAH7817 | 24 | *Poropuntius opisthopterus*[21], *Poropuntius opisthoptera*[3] |
| ***Schistura disparizona*** | ***Schistura disparizona*** | Species | BOLD:ACM0452 | 7 | *Schistura disparizona*[6], *Schistura cf. disparizona*[1] |
| ***Schizothorax gongshanensis***  ***Schizothorax lissolabiatus***  ***Schizothorax nukiangensis*** | ***Schizothorax gongshanensis Schizothorax lissolabiatus Schizothorax nukiangensis*** | species | BOLD:ACP3477 | 280 | *Schizothorax nukiangensis*[226], *Schizothorax lissolabiatus*[37], *Schizothorax gongshanensis*[17] |
| ***Schizothorax wangchiachii*** | ***Schizothorax wangchiachii*** | species | BOLD:AAI0826 | 11 | *Schizothorax wangchiachii*[8], *Schizothorax grahami*[2], *Schizothorax longibarbus*[1] |
| ***Abbottina rivularis*** |  | species | BOLD:ACM1972 | 8 |  |
| ***Abbottina rivularis*** |  | species | BOLD:ACP4217 | 5 |  |
| ***Akrokolioplax bicornis*** |  | species | BOLD:ACB5585 | 75 |  |
| ***Bagarius yarrelli*** |  | species | BOLD:ACH7954 | 6 |  |
| ***Barilius caudiocellatus*** |  | species | BOLD:ACP3873 | 8 |  |
| ***Botia rostrata*** |  | species | BOLD:ABV1876 | 8 |  |
| ***Channa gachua*** |  | species | BOLD:ACP4442 | 21 |  |
| ***Creteuchiloglanis macropterus*** |  | species | BOLD:ACP4817 | 7 |  |
| ***Crossocheilus burmanicus*** |  | species | BOLD:ACB4670 | 5 |  |
| ***Danio shanensis*** |  | species | BOLD:ACP4815 | 12 |  |
| ***Exostoma labiatum*** |  | species | BOLD:ACH6237 | 11 |  |
| ***Gagata dolichonema*** |  | species | BOLD:AAY3858 | 15 |  |
| ***Garra cryptonemus*** |  | species | BOLD:ACB5565 | 48 |  |
| ***Garra salweenica*** |  | species | BOLD:ACP3868 | 58 |  |
| ***Glyptothorax lanceatus*** |  | species | BOLD:ACB4860 | 3 |  |
| ***Glyptothorax longinema*** |  | species | BOLD:ACC0887 | 28 |  |
| ***Glyptothorax zanaensis*** |  | species | BOLD:ACC0607 | 18 |  |
| ***Hemimyzon nujiangensis*** |  | species | BOLD:ACP4597 | 2 |  |
| ***Monopterus albus*** |  | species | BOLD:AAH3279 | 14 |  |
| ***Paramisgurnus dabryanus*** |  | species | BOLD:AAF8881 | 1 |  |
| ***Pseudorasbora parva*** |  | species | BOLD:AAD0138 | 125 |  |
| ***Pteronemacheilus meridionalis*** |  | species | BOLD:ACM0653 | 20 |  |
| ***Rhodeus ocellatus*** |  | species | BOLD:ACP4548 | 12 |  |
| ***Rhodeus ocellatus*** |  | species | BOLD:ACP4547 | 4 |  |
| ***Schizothorax yunnanensis paoshanensis*** |  | species | BOLD:ACP3723 | 33 |  |
| ***Triplophysa stenura*** |  | species | BOLD:ACP4439 | 10 |  |
| ***Pseudexostoma brachysoma*** |  | species | BOLD:ACP3872 |  |  |

Table S5

|  | | | Prior intraspecific divergence (*P*) | | | | | | | |
| --- | --- | --- | --- | --- | --- | --- | --- | --- | --- | --- |
| Model | X | Partition | 0.0599 | 0.0359 | 0.0215 | 0.0129 | 0.0077 | 0.0046 | 0.0028 | 0.0017 |
| simple | 1.5 | Initial | 34 | 34 | 34 | 49 | 49 | 49 | 49 | 49 |
|  |  | Recursive | 34 | 35 | 38 | 49 | 49 | 51 | 59 | 59 |
| JC | 1.5 | Initial | 34 | 34 | 34 | 43 | 43 | 43 | 43 | 43 |
|  |  | Recursive | 34 | 35 | 38 | 45 | 46 | 48 | 56 | 56 |
| K2P | 1.5 | Initial | 34 | 34 | 34 | 43 | 43 | 43 | 43 | 43 |
|  |  | Recursive | 34 | 35 | 38 | 45 | 46 | 51 | 53 | 53 |

Table S6

| Analysis | Clusters (CI) | Entities (CI) | Likelihood_null_ | Likelihood_GMYC_ | Likelihood ratio | Threshold |
| --- | --- | --- | --- | --- | --- | --- |
| Single | 37 (32-41) | 48 (42-58) | 1363.36 | 1374.15 | 21.57*** | -0.004985897 |
| Multiple | 36 (33-38) | 50 (44-66) | 1363.36 | 1376.71 | 26.70*** | -0.01357441 |
|  |  |  |  |  |  | -0.006024188 |
|  |  |  |  |  |  | -0.004315924 |
|  |  |  |  |  |  | -0.003387939 |

Figure S1


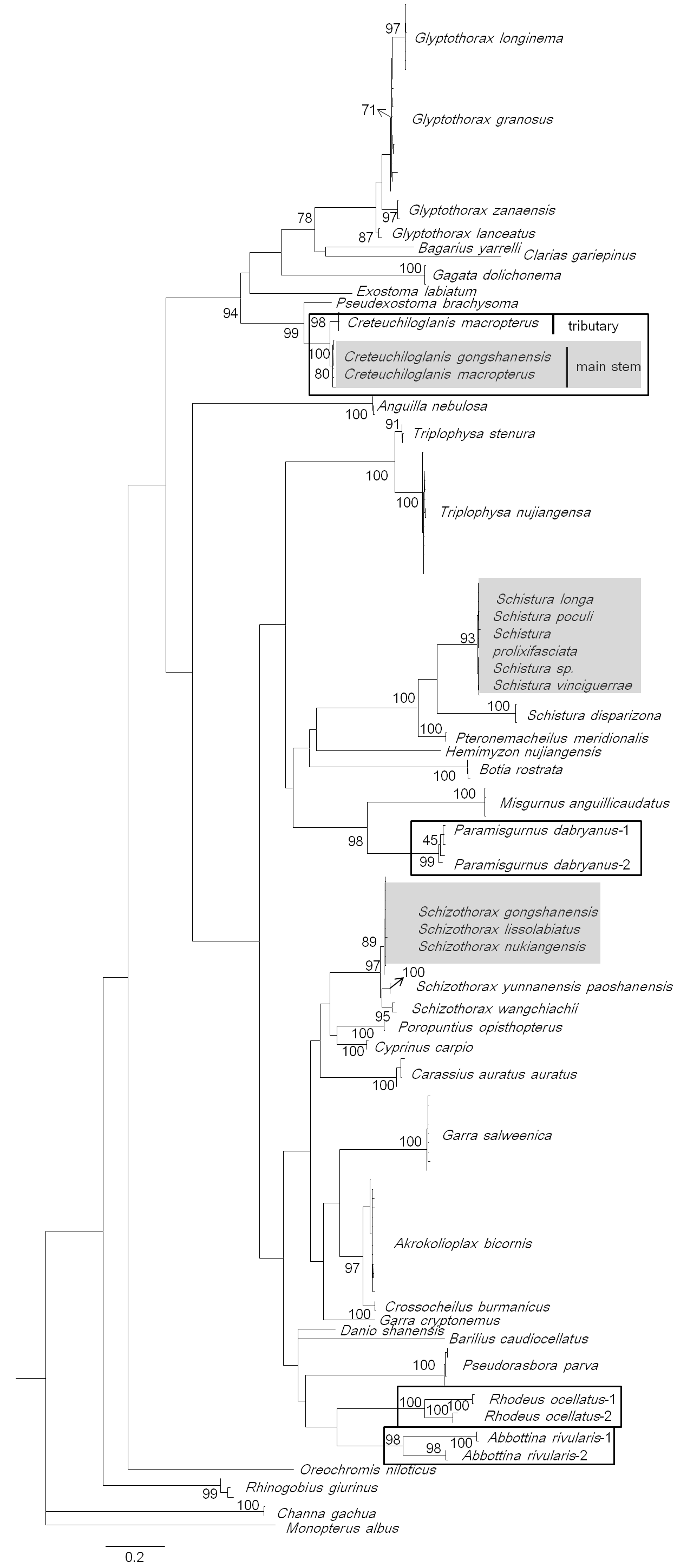


Figure S2


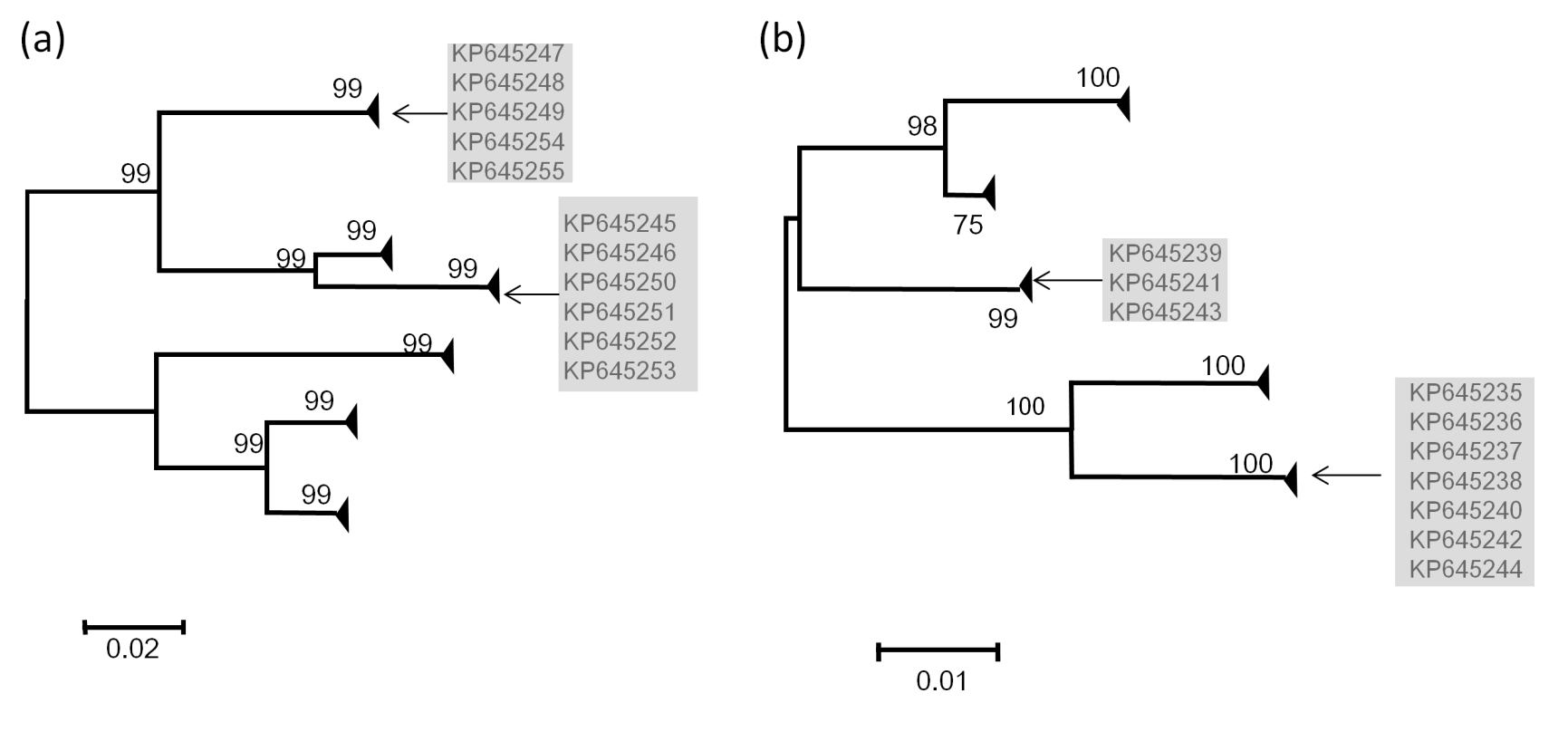

Supplement: Supplementary Information [file srep17437-s1.docx]
